# Druggable binding sites in the multicomponent assemblies that characterise DNA double-strand-break repair through non-homologous end joining

**DOI:** 10.1042/EBC20190092

**Published:** 2020-06-24

**Authors:** Antonia Kefala Stavridi, Robert Appleby, Shikang Liang, Tom L. Blundell, Amanda K. Chaplin

**Affiliations:** Department of Biochemistry, University of Cambridge, Tennis Court Road, Cambridge CB21GA, Cambridgeshire, U.K.

**Keywords:** DNA-repair, multicomponent systems, structural biology, therapeutics

## Abstract

Non-homologous end joining (NHEJ) is one of the two principal damage repair pathways for DNA double-strand breaks in cells. In this review, we give a brief overview of the system including a discussion of the effects of deregulation of NHEJ components in carcinogenesis and resistance to cancer therapy. We then discuss the relevance of targeting NHEJ components pharmacologically as a potential cancer therapy and review previous approaches to orthosteric regulation of NHEJ factors. Given the limited success of previous investigations to develop inhibitors against individual components, we give a brief discussion of the recent advances in computational and structural biology that allow us to explore different targets, with a particular focus on modulating protein–protein interaction interfaces. We illustrate this discussion with three examples showcasing some current approaches to developing protein–protein interaction inhibitors to modulate the assembly of NHEJ multiprotein complexes in space and time.

## Background for NHEJ

Humans use the DNA-damage response (DDR) and DNA-repair pathways to repair the majority of the tens of thousands of DNA lesions that each of their cells experience each day [1]. Amongst the many forms of DNA damage, double-strand breaks are the rarest but most cytotoxic; if left unrepaired, genetic abnormalities, chromosomal instability and cell death may occur [1,2]. Double-strand breaks are repaired by two main mechanisms: Homologous Recombination (HR) and Non-Homologous End Joining (NHEJ). HR progresses through strand invasion on a homologous chromatid, thus restricting HR to mid-S and G2 phases, whereas NHEJ, which tends to be more error-prone, does not require the presence of a sister chromatid and is active throughout the cell cycle [2]. Recent studies have also reported the existence of an alternative-NHEJ pathway (A-NHEJ), which operates when canonical NHEJ (c-NHEJ) is impaired [3]. A-NHEJ uses short-end-microhomology regions, but recent studies indicate that this is not always a requirement [4]. A particular form of A-NHEJ is Microhomology-Mediated End Joining (MMEJ), a mutagenic-repair pathway that requires the presence of microhomology at the DNA ends [5].

**Figure 1:**
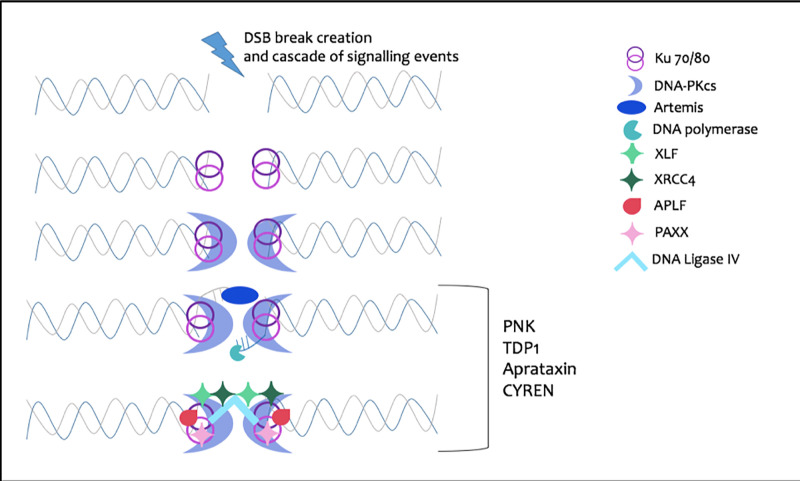
Overview of the NHEJ pathway Activated by a cascade of signalling events as part of the DNA damage response, Ku 70/80 is recruited to the double-strand-break site, forming strong, non-specific interactions with the DNA ends. Ku 70/80 recruits DNA-PKcs, forming the DNA-PK complex. This ‘synapsis’ step facilitates bridging of the two DNA ends together. However, in many cases, the DNA ends require processing prior to ligation, and this is achieved by the exonuclease Artemis, while DNA synthesis is mediated by the DNA polymerases μ and λ. Finally, ligation is performed by the LigaseIV, which is in complex with the XRCC4 scaffolding factor. PNKP prepares the DNA ends by addition and removal of phosphate groups. Several other scaffold proteins also support the ligation step, primarily XLF, which is known to form filaments with XRCC4 to support the ligation process. Other scaffold factors include APLF and PAXX. Several other proteins have been reported to participate in the NHEJ pathway, including aprataxin, TDP1 and CYREN.

The initial stage of NHEJ requires binding of the heterodimeric Ku70/80 to the free-DNA ends, forming strong, non-specific, non-covalent interactions with the DNA phosphate backbone [6]. Ku 70/80 in turn recruits the DNA-PK catalytic subunit, DNA-PKcs, forming the DNA-PK complex. DNA-PK mediates the synapsis bridging of the two DNA termini and orchestrates subsequent protein–protein interactions (PPIs) [7]. Subsequently, non-compatible or resection-dependent DNA ends are processed by the recruitment of the nuclease Artemis through interactions with DNA-PKcs. In cases where DNA synthesis is required, this is performed by the polymerases μ and λ in a template-dependent manner, while polymerase μ can also act in a template-independent manner [4]. Following end processing, ligation of DNA ends, either blunt or incompatible, is performed by the DNA Ligase IV (LigIV) in complex with the scaffolding factor, XRCC4 [8]. Polynucleotide kinase/phosphatase (PNKP) also assists ligation through the creation of 5-phosphate and 3-hydroxyl ends [9]. Additional scaffold proteins are recruited to assist in complex stability, including XLF, shown to form filaments *in vitro* with XRCC4 to stabilise DNA ends for ligation, while super-resolution microscopy studies have reported the formation of long XLF-XRCC4-LigIV filaments [10–12]. PAXX, a factor recently discovered by our group, aprataxin and PNK-like factor (APLF) have supporting roles the assembly of NHEJ complexes and pathway progression [13,14]. Several accessory factors participate to support NHEJ, examples being aprataxin, tyrosyl DNA phosphodiesterase 1 (TDP1) and CYREN [15] (Figure 1).

In this review, we give an overview of the role of NHEJ in cancer progression and therapy resistance, its application using the concept of synthetic lethality, and the efforts previously carried out in targeting individual NHEJ components as anti-cancer therapeutics. We discuss not only targeting active sites but also protein–protein interaction (PPI) inhibition in NHEJ as a promising approach.

## Impairments in NHEJ and its relation to disease

DNA within humans is constantly exposed to a potentially damaging environment and DDR mechanisms act in order to counteract this. These DNA repair processes act in parallel, adapting to the specific type of damage and stage of the cell cycle. Not all mutations contribute equally to carcinogenesis and have thus been divided into ‘driver’ (driving cancer progression) and ‘passenger’ mutations (are neutral) [16]. Certain mutations in NHEJ components that lead to pathway deregulation have been reported as drivers for carcinogenesis and cancer progression [17]. An overactive, error-prone NHEJ can provide a survival advantage for cancer cells compared with normal cells, as they manage to rapidly repair endogenous or exogenous DNA damages [4]. In cancer patients, most observed mutations are associated with up-regulation of NHEJ proteins but there are cases characterised by decreased expression [17]. For example, colorectal tumour profiles have shown increased Ku levels and elevated DNA-binding activity [18], while studies focusing on prostate cancer tissues, non-small cell lung carcinomas (NSCLC) and hepatocellular carcinomas (HCC), have all shown elevated DNA-PKcs expression [19–21]. XLF overexpression has been reported in Human Papilloma Virus (HPV) positive head and neck squamous cell carcinomas (HNSCCs) [22], while certain prostate tumour types have reported high levels of LigIV [23]. Single-nucleotide polymorphisms in NHEJ factors have also been reported in a few cancer patients and linked to carcinogenesis [17].

Importantly, an overactive NHEJ machinery has been linked to chemo- and radio-therapy resistance, as overexpression of core components allows efficient repair of double-strand breaks created by those therapies [17]. For example, increased DNA-PKcs levels enhance therapy resistance in cervical and ovarian cancers, and cisplatin therapy in glioma [24–26]. Several cell studies on colorectal cancer cells and oral cancer stem cells have also linked increased LigIV and XLF expression respectively, to radio-resistance [27,28]. On the contrary, cervical carcinomas with low Ku70 expression levels showed radiosensitivity whereas NSCLC patients with lower DNA-PKcs levels responded better to therapy [29,30].

It therefore becomes apparent that NHEJ could be manipulated in the area of cancer therapy in an effort to combat resistance. Inhibiting NHEJ in conjunction with radio- or chemo-therapies could reduce the tumour cells’ ability to repair therapy-induced double-strand breaks. The lesions are more detrimental to the survival of neoplastic cancer cells than their surrounding normal cells, so reducing off-target effects and the therapeutic quantities required [17]. However, absence of NHEJ can result in signalling for activation of alternative, sometimes highly mutagenic pathways that are able to restore DNA repair in cancer cells and prolong their survival [4,31]. Targeting of NHEJ therefore requires careful examination of its temporal and spatial organisation to minimise the probability of a rebalancing act, as discussed below.

## Previous successes and the concept of synthetic lethality

Over the years there has been a great interest in understanding and targeting DNA damage response, with a focus on poly (ADP-ribose) polymerase (PARP) enzymes. PARP recognises single-strand breaks and mediates the recruitment of DNA repair factors [32]. By binding and catalysing PARylation events, PARP eventually auto-PARylates, which allows its release from DNA [32]. Preventing this autoPARylation event abrogates PARP release from DNA leading to the progression of the single-strand break to a double-strand break, highlighting PARP, as an attractive drug target [32,33]. Three PARP-1 inhibitors are already available in the clinic to treat BRCA-1 and BRCA-2 deficient breast and ovarian cancers through achieving synthetic lethality [34]. Synthetic lethality takes place when simultaneous loss of two genes leads to cell death, and has now become an attractive therapeutic tactic for cancers presenting genetic defects in certain components by inhibiting a protein that acts as a survival mechanism for them, leading to cell death [34].

Here, we will instead focus on the different approaches to target NHEJ, where, despite its importance as the main double-strand break repair pathway, limited success has been recorded. The majority of research thus far, apart from the few exceptions mentioned below, has been limited to computational, *in vitro* and cell studies. As aforementioned, targeting NHEJ can be critical in battling therapy resistance, while NHEJ could also be manipulated to achieve synthetic lethality. Indeed, a recent study supported the contention that an overactive-NHEJ acts as a mechanism of resistance in PARP1-FANCA synthetic lethality models, while its inhibition actually prevented NHEJ-driven resistance to the chemotherapeutic agent mytomycin C in those models [35].

## Targeting individual NHEJ proteins

### DNA-PKCs kinase active site

**Figure 2:**
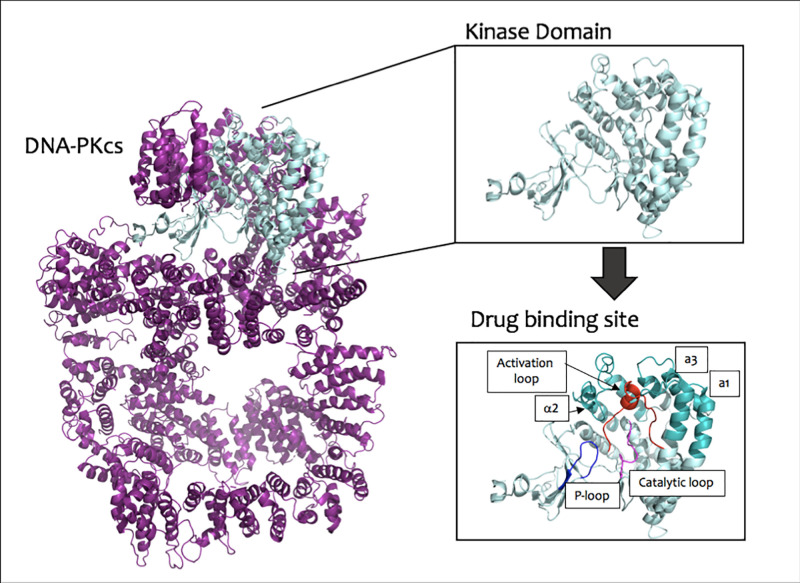
DNA-PKcs structure, highlighting the kinase domain and predicted DNA-PK inhibitor drug-binding site Apo-DNA-PKcs structure is shown in purple with the kinase domain highlighted in light blue (Sharif et al., (2017), PDB: 5W1R [36], equivalent to PDB: 5LUQ coordinates [37]). Insets display the kinase domain (residues 3676-4100) and the drug-binding site, with the key features labelled (see text for further discussion).

The greatest focus by far has been on DNA-PK, as the kinase active site of DNA-PKcs is an attractive and more approachable target. It has however still been a major challenge to identify inhibitors of DNA-PKcs that have good selectivity to prevent its kinase enzymatic activity but do not inhibit structurally related kinases (Figure 2).

Many of the specific DNA-PK inhibitors that target the ATP-binding site of the kinase domain are limited by poor solubility and high metabolic lability, with the most important strategy being to develop compounds based on existing drugs [38,39]. The current drugs known to target DNA-PK are summarised in Table 1.

**Table 1:** A selection of compounds that display DNA-PK inhibition

Compound name	IC_50_	Formula/Molecular weight (gmol^−1^)	Solubility	Cancer example type	Mechanism	Year	Reference(s)
Wortmanin	5 nM	C_23_H_24_O/ 428.4	DMSO	N/A	Lys802 Irreversible covalent modification	1993	[40]
LY294002	0.5-1.4 μM	C_19_H_17_NO_3_/307.4	DMSO, ethanol and dimethyl formamide to 16 mg/ml	N/A	ATP-competitive inhibitor	1994	[41]
IC86621	120 nM	C_12_H_15_NO_3_/221.3	DMSO: ≥10 mg/ml	Promising results xenografts	ATP-competitive inhibitor	2003	[42,43]
IC87361	34 nM	C_19_H_17_NO_4_/323.3	DMSO	Promising results xenografts	ATP-competitive inhibitor	2003	[42,44]
Vanilin	1.5 mM	C_8_H_8_O_3_/152.2	Water soluble 10 g/l	Solid tumours	Lys Irreversible covalent modification	2003	[45]
NU7441	13 nM	C_25_H_19_NO_3_S/413.5	DMSO : 14.29 mg/ml	Solid tumours, liver cells, non-small cell lung carcinoma	ATP-competitive inhibitor	2004	[46,47]
NU7026	0.23 μM	C_17_H_15_NO_3_/281.31	DMSO : 2.9 mg/ml	Solid tumours, liver cells, non-small cell lung carcinoma, gastric cancer	ATP-competitive inhibitor	2004	[48]
PX866 Sonolisib (wortmannin analogue)	0.1–1 nM	C_29_H_35_NO_8_/525.6	DMSO/ethanol at 200 mg/ml; very poorly soluble in water	Solid tumours, glioblastoma, melanoma, prostate, advanced BRAF-mutant cancers and non-small cell lung cancer	Lys802 Irreversible covalent modification	2004	[49–52]
PWT-458 (pegylated-17 hydroxywortmannin)	1–200 nM	C_23_H_26_O_8_/5430.4 (pegylated)	Soluble in 1:9 EtOH:PBS (pH 7.2) (∼0.1 mg/ml), ethanol (∼0.15 mg/ml), DMSO (∼2.5 mg/ml) and DMF (∼3 mg/ml).	Glioma, non-small cell lung cancer, renal cell carcinoma and solid tumours	Lys802 Irreversible covalent modification	2005	[53]
PI103 hydrochloride	2 nM	C_19_H_16_N_4_O_3_.HCl/384.8	DMSO : 4.1 mg/ml	Tumour growth malignancies	ATP-competitive inhibitor	2006	[54]
SF1126 Semafore	7-9 μM	C_39_H_48_N_8_O_14/_852.8	Water soluble	Glioma, prostate, non-small cell lung cancer, colorectal and breast cancer	ATP-competitive inhibitor	2008	[55]
KU 0060648	8.6 nM	C_33_H_34_N_4_O_4_S/582.7	DMSO: 1 mg/ml	Hepatocellular carcinoma	ATP-competitive inhibitor	2012	[56]
VX-984	88 nM	C_23_H_21_D_2_N_7_O/415.49	DMSO : 10 mg/ml	Advanced solid tumours, lymphomas	ATP-competitive inhibitor	2016	[57]
LY3023414	4.24 nM	C_23_H_26_N_4_O_3_/406.5	DMSO : 50 mg/ml	Solid tumours	ATP-competitive inhibitor	2016	[58]
CC-115	13 nM	C_16_H_16_N_8_O/336.4	DMSO : ≥32 mg/ml	Glioblastoma, prostate cancer	ATP-competitive inhibitor	2017	[59]
M3814 Nedisertib	<3 nM	C_24_H_21_ClFN_5_O_3_/481.9	DMSO : 100 mg/ml H_2_O : <0.1 mg/ml	Small cell lung cancer, rectal cancer, bone marrow, acute myeloid leukaemia	ATP-competitive inhibitor	2017	[60,61]
AZD7648	0.6 nM	C_18_H_20_N_8_O_2_/380.4	DMSO : 5 mg/ml	Advanced malignancies, non-small cell lung cancer	ATP-competitive inhibitor	2019	[62]

Wortmannin, one of the first identified inhibitors of DNA-PK and a naturally occurring compound, is a potent non-competitive irreversible inhibitor of PI3K, PIKK and DNA-PK [39,63]. Wortmannin does, however, display substantial *in vivo* toxicity and is thus unsuitable for systemic therapeutic applications. Modifications of Wortmannin have been designed with increased selectivity and extended half-lives [49,64]. One example is PWT-458, a pegylated 17-hydroxywortmannin derivative, which is water soluble and shows improvements in both drug stability and *in vivo* pharmacokinetic parameters [53,65].

Another early described inhibitor is 2-(4-morpholine)-8-phenyl-4 hydrogen-1-benzo-4 ketone (LY294002) [41]. LY294002 has a broad inhibitory role not only for DNA-PK but also for other protein kinases [66,67]. LY294002 does, however, result in potent anti-tumour and anti-angiogenic activity *in vivo* [68,69]. SF1126 is a covalent conjugate of LY294002, which has been evaluated in numerous animal tumour models and shown to inhibit colorectal cancer growth [55,70]. This compound led to the evolution of several potent DNA-PK inhibitors including IC86621 and IC87361, of which the latter is 50-fold more selective for DNA-PK than for other kinases [42,44]. Ly294002 was also used as the starting point for the synthesis of many further compounds: of these NU7026 (2-(morpholin-4-yl)-benzo[h]chromen-4-one) was 6-fold more potent and 70-fold more selective for DNA-PK [48]. Nevertheless, although *in vitro* studies were promising, pre-clinical results showed that the drug is quickly cleared from circulation [71].

Furthermore, several compounds display mixed activity against DNA-PK, including caffeine [72], vanillin [45], and two compounds that are currently in clinical development and act against mTOR and DNA-PK, LY3023414 and CC-15 [58,59]. Newer generation specific DNA-PK compounds include VX-984 and M3814 that are now in clinical development [57,60,61]. Preliminary results indicate that VX-984 enhances radio-sensitivity of brain tumour xenografts and could help in management of glioblastoma cells [57]. Even though M3814 has shown limited efficacy as a single agent in ovarian cancer, together with pegylated liposomal doxorubicin it showed enhanced activity [61]. The newest compound is the potent and highly selective DNA-PK inhibitor AZD7648, developed by AstraZeneca late last year. In the publication describing AZD7648, the authors also explore the potential for DNA-PK inhibitors as combinatorial agents with other DNA damage response-targeted agents [62]. They demonstrate that AZD7648 enhances the efficacy of both ionising radiation, doxorubicin and in combination with olaparib, a PARP inhibitor currently approved for breast and ovarian cancers [62]. These combinations have now progressed to clinical trial (trial identifier: NCT03907969).

There are also additional DNA-PK inhibitors not discussed within this review article. However, it can be concluded from Table 1 that those identified thus far generally act by interfering with the ATP-binding site of the DNA-PKcs kinase domain with differing degrees of selectivity, potency and reversibility [73,74]. Targeting the kinase site is partly why developing such compounds to be specific inhibitors for DNA-PK is so challenging. It is, therefore, paramount for continual improvement of such compounds that the structure and mechanism of the DNA-PK holoenzyme in NHEJ must be fully understood. To date, no high resolution structural information of DNA-PK inhibitors have been shown. Structural information of DNA-PK and its NHEJ binding partners will allow us to assess drug-binding sites and mechanisms other than the kinase domain.

### Ku 70/80 DNA-binding site

**Figure 3:**
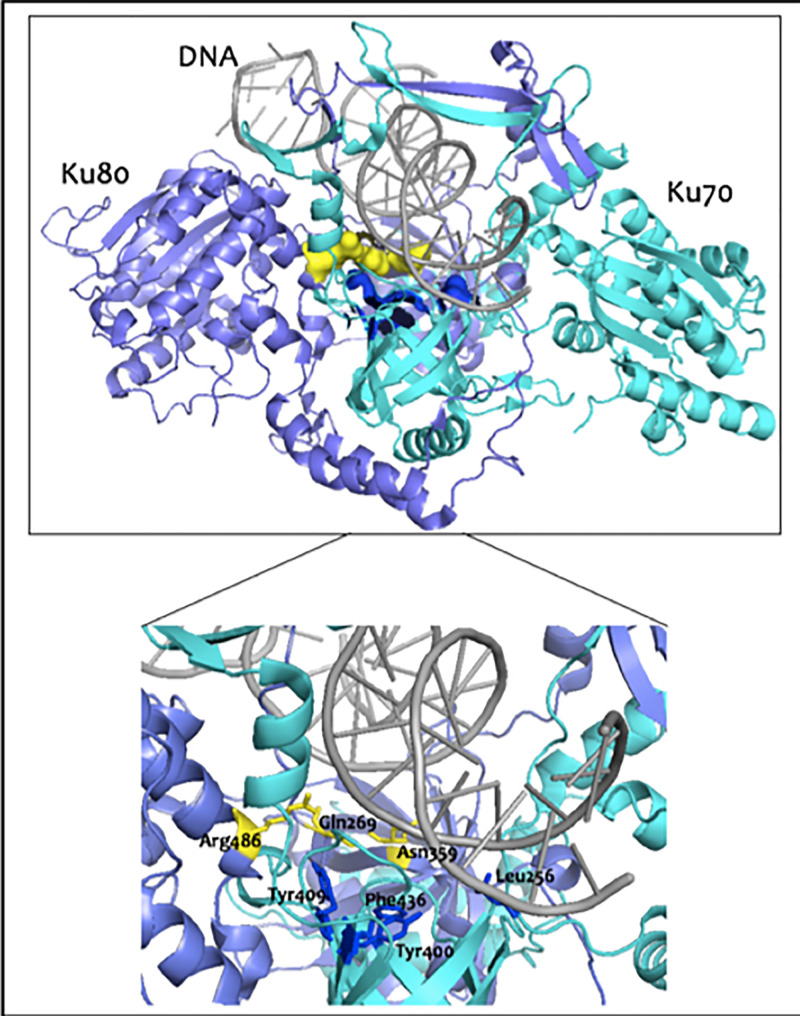
Illustration of the binding pocket identified for Ku 70/80 The pocket was identified using Surflex-Dock software. The pocket, shown as a surface (dark blue and yellow), is in close proximity to the DNA-binding ring-like structure. Ku 70 is show in cyan, Ku 80 in light purple and DNA in grey (Walker et al., 2001; PDB: 1JEY [6]). A closer view of the pocket shows that it comprises seven residues: four belong to Ku70 and three to Ku80. Residues from Ku70 are shown in dark blue and those from Ku80 in yellow. Residues labelled as in Weterings et al., 2016 [75].

Recently, a putative binding pocket for Ku 70/80 was identified through *in silico* pocket-based drug discovery, and ‘idealised ligands’ were generated and docked against the binding pocket using the Surflex-Dock software [75]. The latter predicted the presence of a binding pocket comprises seven amino acids located at the interface between Ku70 and Ku80 and close to the ring where DNA binds (see [75] for full visualisation of pocket) (Figure 3). The authors showed that a compound, known as Compound L, could bind in this pocket with micromolar affinity, and biochemical studies showed it was able to disrupt DNA binding and DNA-PKcs recruitment and activation *in vitro*, while cell-based studies also linked increasing concentrations of this compound with radiosensitivity to SF-767 and glioblastoma human cell lines [75]. As the authors report, Compound L is the only known inhibitor of Ku70/80 and requires further development to become a potential lead. However, targeting NHEJ at its initial DNA-binding stage could promote signalling processes that would drive activation of an alternative repair pathway. Indeed, genetic studies examining the absence of several core NHEJ factors showed that in Ku80-null cells microhomology-joining events, resembling those of A-NHEJ, were observed and double-strand break repair was still taking place [76]. What is more, the DNA-PK complex seems to have an inhibitory effect on binding of A-NHEJ factors, such as PARP-1, to double-strand breaks [77]. This suggests that inhibiting the NHEJ pathway at a very early stage could prove inefficient in inhibiting double-strand break repair and in battling radio- and chemotherapy resistance.

### Ligase IV DNA-binding site

**Figure 4:**
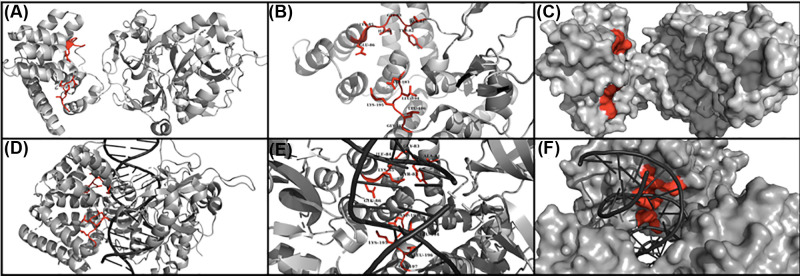
Orthosteric drug targeting of DNA Ligase IV Illustration of the catalytic region of DNA Ligase IV showing two conserved regions (81-86; 193-197) which were targeted in Srivastava et al., 2012 (red) [79]. (**A**) DNA Ligase IV apo state (Ochi et al., 2013; PDB: 3W5O [83]). (**B**) DNA Ligase IV zoomed in on conserved residues in the apo state (Ochi et al., 2013; PDB: 3W5O [83]). (**C**) Molecular surface of the DNA Ligase IV apo state (Ochi et al., 2013; PDB: 3W5O [83]) (**D**) DNA Ligase IV DNA-bound state (Kaminski et al., 2018; PDB: 6BKG [84]). (**E**) DNA Ligase IV zoomed in on conserved residues in the DNA-bound state. (Kaminski et al., 2018; PDB: 6BKG [84]). (**F**) Molecular surface of DNA Ligase IV in the DNA-bound state. (Kaminski et al., 2018; PDB: 6BKG [84]).

DNA Ligase IV has also attracted attention as a target for inhibiting NHEJ by virtue of its role as the only ligase of the system; LigIV therefore exhibits a non-redundant mechanism of functional control over ligation. Echoing arguments above, abrogating ligation as opposed to synapsis is thought to have a reduced likelihood of invoking alternative redundant double-strand-break pathways [4]. All published drug-discovery approaches targeting LigIV so far have been of an orthosteric nature (Figure 4). Chen et al. (2008) identified L189 as a competitive inhibitor of LigIV, developed from the virtual screening against the DNA Ligase I DNA-binding domain; however, this molecule also showed undesirable broad-spectrum inhibition of DNA Ligases I & III [78]. Srivastava et al. (2012) followed by using a rational design approach based on a 3D model of LigIV, generated using templates of DNA-binding domains from other ligases. They focused on two spatially conserved putative DNA-binding regions of LigIV and developed the DNA-binding inhibitor SCR7, a derivative of L189, which was initially suggested to be more selective for LigIV [79]. However, a more recent publication reported stronger inhibition by SCR7 of LigI and LigIII than LigIV in ligation assays [80]. Recent investigations have highlighted the potential of treating cells with SCR7 to increase the efficiency of CRISPR–Cas9-mediated gene editing by inhibiting NHEJ and favouring HR [81,82]; however, the mechanism of action is still unclear, indicating that more research is required on this area.

### *Polymerases* μ and λ

Targeting the active site of polymerases and stalling DNA synthesis could also be a useful approach. This is ideal for double-strand-break repair pathways, because NHEJ and HR use different polymerases, which could potentially minimise off-target effects [85]. However, early studies on vertebrate cells deficient in pol μ and/or λ, showed minimal-to-no radiosensitivity [86]. More recent studies, however, indicate that even though absence of either of the two polymerases does not significantly impair NHEJ, their simultaneous loss leads to high radiosensitivity in cells, with similar levels to those observed upon complete loss NHEJ [87], potentially revealing a new area for drug discovery. To the best of our knowledge, there are no published studies of inhibitors against the active sites of the two NHEJ polymerases. However, recent studies examining the interactions of polymerase λ with XRCC4, XLF and PAXX showed that they promote its recruitment to damage sites and control its function; this could shed light in using these interactions as drug targets [88].

### XRCC4

Molecular dynamics simulations based on a screen from the traditional Chinese Medicine Library (TCM) have also supported the use of salvianolic acid B, lithospermic acid and 2-*O*-feruloyl tartaric acid as inhibitors of XRCC4 activity [89]. However, these studies are rather preliminary and the potential effect of these agents needs to be tested *in vitro* and subsequently *in vivo* [89]. A recent study also reported that perfluorodecanoic acid (PFDA) targeting XRCC4 was able sensitise gastric adenocarcinoma cell lines to chemotherapy, but such targeting was on the mRNA expression level rather than targeting the protein as on all the examples above [90].

## Targeting protein–protein interactions in NHEJ

PPIs are fundamental for many cellular processes involved in mediating and regulating signalling processes and in pathway progression. However, they have often been described as undruggable, mainly because the interfaces between two globular proteins are usually large and flat, limiting their use at drug targets [91,92]. Indeed, inhibitors targeting such interfaces exist for less than 0.01% of known PPIs [93]. However, as Jubb et al. (2015) have commented, when the interface involves concerted folding and binding of a previously unfolded polypeptide region on to a preformed globular structure, the pockets are usually deeper and are thus more suitable for drug discovery [91]. In this case, the loss of entropy when the ligand binds a deep pocket is compensated by displacing the water molecules into the aqueous environment, where they gain entropy. The entropic gain becomes even more favourable if the pockets feature a juxtaposition of polar and lipophilic interactions, further limiting the rotational entropy of the bound waters to the apo-state. These features have been used to develop a server to map hotspots for ligand binding within a protein, and these can be used to identify druggable sites at protein–protein interfaces [94]. Numerous other druggability predictions have been developed, integrating structural and chemical information to predict pocket druggability based on approaches including machine learning, as with the DoGsite scorer software [95] or linear discriminant analysis is in the case of PockDrug [96].

The druggable binding sites at protein–protein interfaces can be quickly explored experimentally using fragment-based approaches [97,98]. Fragments bind only at hotspots unless the concentrations are very high. Furthermore, the availability of libraries of <1000 fragments allows efficient exploration of a large chemical space to identify hits that can be elaborated into leads for drug discovery. X-ray crystallography has until recently been the gold standard for structure-based drug discovery. This is due to its ability to obtain high-resolution structures that reveal the electron density of small inhibitors or that of water molecules. Such structure-guided fragment-based approaches have been used by our group over nearly two decades to identify druggable sites and new leads for targeting DNA double-strand-break repair through HR, for example targeting the BRCA2 binding site in RAD51 [99,100]. More recently, we have been using these techniques to target protein–protein interfaces in NHEJ proteins, as these form many different types of interactions within the space and time of the double-strand-break repair process [84,85].

With regards to NHEJ, so far, we have seen many crystallographic structures. A few domains have also been resolved using nuclear magnetic resonance (NMR) techniques, with the prime example being the Ku80 C-terminal domain [101]. Cryo-EM has defined low resolution structures of DNA-PK and other components of NHEJ, but has over the past few years undergone a ‘resolution revolution’ allowing it to produce structures where fragments can be observed [102]. Although flexibility, particularly of DNA-PKcs, has resulted in medium-resolution cryo-EM models from many groups working on NHEJ proteins [36,101,103], models at resolutions approaching 3Å of human DNA-PKcs and DNA-PK have now been achieved (Chaplin A.K., Hardwick S.W., Liang S, Kefala Stavridi A., Hnizda A., Chirgadze D.Y., Cooper L., De Oliveira T.M., Blundell T.L., unpublished). Indeed, cryo-EM is beginning to provide us with the ability to examine protein–protein interfaces of large protein complexes, such as those that have been observed or are hypothesised to exist in NHEJ. Moreover, new developments in cryo-EM, including advanced sample preparation protocols, state-of-the-art cryo-EM detectors and data processing software, are now able to retrieve high enough resolutions with the flexible, multicomponent systems of NHEJ to allow the visualisation of inhibitor densities.

Using a structure-guided drug discovery approach, by combining computational and experimental studies, we should be able assess the drugabbility of PPIs at different stages of the NHEJ pathway, which could be more fruitful than targeting individual components, as the pathway itself is mainly mediated by those interactions rather than individual components. The unusual nature of certain interactions could also assist in achieving specificity and minimise off-target effects, in contrast to targeting common active sites.

### Ku 80–XLF interaction

**Figure 5:**
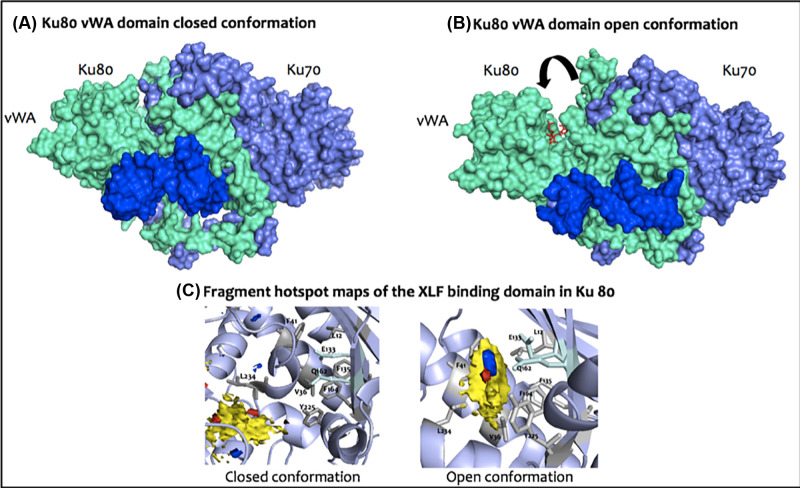
Illustration of the Ku80-XLF interaction and its potential druggability (**A**) Crystal structure of Ku 70/80 in complex with DNA, showing a closed conformation of the Ku 80 vWA domain (Walker et al., 2001; PDB: 1JEY [6]). Ku 70 is shown in slate and Ku80 in light green. The DNA visible at this angle (dark blue) corresponds to the hairpin part of the duplex DNA used for this study. The latter is used to block Ku from sliding off for crystallisation (**B**) Crystal structure of Ku 70/80 in complex with DNA and a 13 -amino acid peptide (density of only eight amino acids was modelled) of the C-terminus of XLF containing its Ku Binding Motif (KBM) (red), showing a conformational change of the Ku80 vWA domain on binding KBM (indicated by arrow), and revealing a buried, deep pocket observed (Nemoz et al., 2018; PDB: 6ERG [104]). Ku 70 is shown in slate and Ku80 in light green. As observed in (A), the DNA visible at this angle corresponds to the hairpin part of the duplex DNA used for this study. (**C**) Fragment hotspot maps of Ku80 in the ‘closed’ and ‘open’ conformations show that in the open conformation a clear hotspot for fragment binding is observed in the XLF binding pocket, which can act as a stepping-stone for structure-based drug discovery.

Nemoz et al. (2018) have determined two crystal structures of Ku 70/80 in complex with peptides containing the Ku binding motif of XLF, one of 19 amino-acid and the other of 13 amino-acid, solved to 2.8 and 2.9Å resolution, respectively [104]. These complexes exhibit a large outward movement resulting from a conformational change in the vWA domain of Ku80, which reveals an allosteric protein-peptide binding site for XLF binding (Figure 5A). Given that only short peptides from XLF C-terminus were used, it is unclear as to whether XLF undergoes extensive conformational changes, for example whether the complete flexible C-terminal tail of XLF folds into a more organised state. The open XLF-binding pocket is deep, and is composed of aromatic and hydrophobic residues surrounded by a few polar ones (Figure 5B). Unpublished work in our group has already examined the XLF-binding pocket in the closed and open states and identified the latter as a fragment hotspot, which could be utilised as a future drug interaction site (Figure 5C). An advantage in exploiting this interaction in drug discovery is that, as argued above, targeting the NHEJ upon establishment of the DNA-PK complex minimises activation of alternative ‘rescue’ double-strand-break repair pathways. Indeed, independent studies, have associated XLF absence with radiosensitivity and NHEJ impairment [10,76,105], while, importantly, mutations in the Ku binding motif of XLF resulted in impairments in XLF recruitment to double-strand-break sites and distortions in XLF-XRCC4 filament formation [104]. Using small-angle X-ray scattering (SAXS), Nemoz et al. (2018) demonstrated that this open conformation exists in equilibrium with the closed one, and XLF binding stabilises it further [104]. It should be noted that targeting this PPI could indeed prove challenging, given the unknowns with regards to the time, space and percentage equilibrium at which the open conformation exists. It will also depend on whether a small compound would be able to exert its effect within this binding pocket that is not always exposed and be able to stabilise this open conformation. However, inducing the opening with a 13 amino acid peptide, in the absence of the full-length XLF, gives hope that this site could be druggable.

### LigIV–Artemis interaction

**Figure 6:**
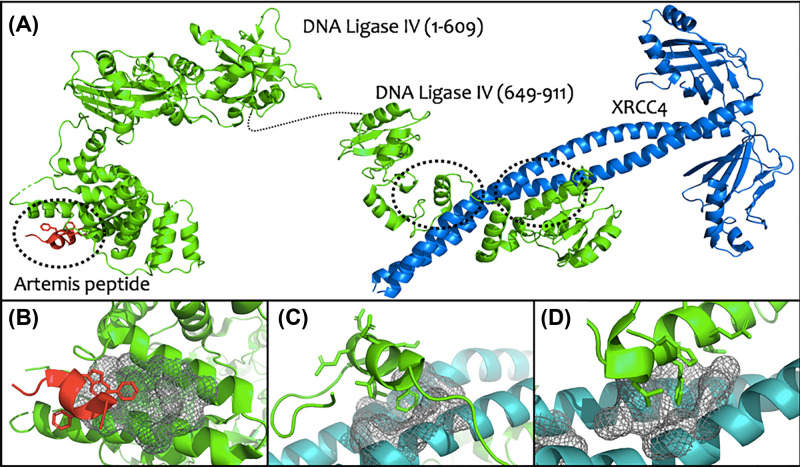
The structural basis of LigIV PPIs with NHEJ components (**A**) The crystal structure of DNA Ligase IV 1-609 (green) in complex with an Artemis peptide (red) corresponding to residues 485-495 (Ochi et al., 2013; PDB: 3W1B [83]) and the crystal structure of the DNA Ligase IV tandem BRCT repeats (649-911) with inter-BRCT linker peptide in complex with an XRCC4 dimer (blue) (Wu et al., 2009; PDB: 3II6 [106]). PPI interfaces ringed. (**B**) A druggable pocket at the interface between DNA Ligase IV and an Artemis peptide (Ochi et al., 2013; PDB: 3W1B [83]). (**C**) The N-terminal helix of the inter-BRCT linker HLH motif docks into a druggable pocket on XRCC4 (Wu et al., 2009; PDB: 3II6 [106]). (**D**) Two helices from the BRCT2 domain dock into a druggable pocket on the opposing face of the XRCC4 coiled coil (Wu et al., 2009; PDB: 3II6 [106]).

In the case of LigIV, there are several interacting partners whose association could be modulated in order to disrupt the recruitment of LigIV to the NHEJ complex (Figure 6A). One of these is the LigIV–Artemis interaction. LigIV recruitment to the double-strand break is thought to be regulated in part by its interaction with the Artemis nuclease C-terminal region. The crystal structure of the catalytic region of LigIV in complex with an Artemis peptide has been solved to 2.4 Å resolution [83] and demonstrates that the predicted region of Artemis undergoes concerted folding and binding, forming a three helical bundle upon interaction with a pocket on the surface of the LigIV DNA-binding domain. Targeting the Artemis-binding pocket on LigIV (Figure 6B) has physiological relevance in potentially disrupting NHEJ complex formation, and recent unpublished findings in our group suggest this pocket is highly druggable. Combinatorial druggability predictions of the aforementioned crystal structure suggest that the Artemis-binding site on LigIV has a propensity for binding drug-like molecules, particularly as it is preformed, promoting ligand-accessibility.

### LigIV–XRCC4 interaction

During ligation, LigIV binds XRCC4 [107,108] through interactions between the LigIV tandem BRCT domains and the inter-BRCT linker region, comprising a helix-loop-helix (HLH) motif, with the XRCC4 coiled coil. The structural basis for this interaction is well defined by crystallographic structures [106,109], showing that the N-terminal helix of the HLH motif and a helix from the BRCT2 domain dock into pockets on opposing faces of the XRCC4 coiled-coil (Fig 6C,D). Unpublished computational analyses from our group suggest both pockets are highly druggable but are likely to prove challenging targets due to the extensive helical rotation of XRCC4 that occurs upon BRCT2 binding [106], indicating that these pockets are likely cryptic in the apo-state. Disrupting the interaction between LigIV and XRCC4 through modulation of the PPI interface is therefore a challenging prospect, and design of a successful inhibitor might instead focus on stabilising the pre-rotational state of XRCC4 to prevent the conformational change associated with LigIV binding. Stabilising the unbound LigIV BRCT domains was an approach taken recently by Menchon and colleagues where they used virtual screening against the LigIV C-terminal clamp domain resulting in the discovery of molecule #3101, which they showed inhibited LigIV–XRCC4 interaction *in vitro* [110], thus presenting a promising future route for the development of an allosteric NHEJ inhibitor.

## Concluding remarks

**Figure 7:**
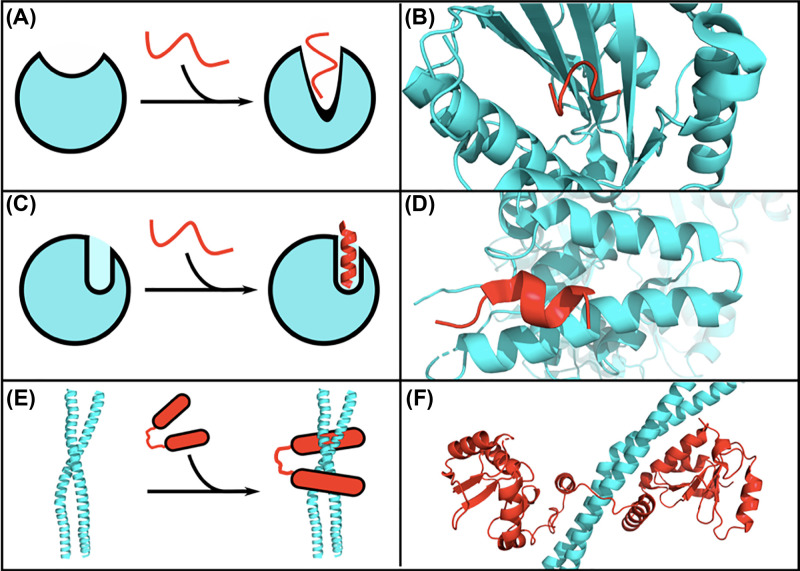
Subtypes of PPI interfaces involving concerted folding and binding in NHEJ complex assembly (**A**) Interface between a globular protein and a flexible peptide, in which binding of the flexible peptide is facilitated by a conformational change in the globular protein to form a deep pocket. (**B**) Example from the interaction interface of Ku80 (cyan) and the Ku-binding motif of XLF (red) (Nemoz et al., 2018; PDB: 6ERG [104]). (**C**) Interface between a globular protein with a preformed pocket and a flexible peptide that undergoes concerted folding and binding. (**D**) Example from the interaction interface between DNA Ligase IV (cyan) and a peptide of Artemis (red) (Ochi et al., 2013; PDB: 3W1B [83]). (**E**) An interface between a coiled coil and a flexible region connecting globular protein domains that undergoes induced folding and binding to form a ‘clamp’. (**F**) interface between the XRCC4 coiled coil (cyan) and the C-terminal BRCT repeat region of DNA Ligase IV (red) (Wu et al., 2009; PDB: 3II6 [106]).

Despite the importance of NHEJ both in the normal function of cells but also as a driver for carcinogenesis and therapy resistance, there are still many unknowns, not only in the structures and functions of these proteins but also in drug-discovery developments. What is certain, however, is that many of these proteins are multi-faceted in character, forming various types of interactions with different NHEJ components, some of which may be present at allosteric binding sites that have not yet been considered fully but could act a further stepping-stone in the search for new drug molecules. To illustrate this, we summarise the PPIs discussed above into three categories (Figure 7). This alone gives us an indication of the array of different types of PPIs that exist during the space and time of NHEJ, and hence the opportunity to specifically target these. Being able to manipulate different points of the NHEJ pathway gives a potential advantage in gaining specificity over simply targeting active or DNA-binding sites. Further down the line, it could even result in the development of personalised therapies, given the profile of patients can vary with regards to NHEJ related defects (reviewed in more detail in Sishc et al. (2017) [17])

## Summary

- Despite its importance in maintaining genomic stability, NHEJ is a driver of carcinogenesis and anti-cancer therapy resistance for many tumour types.
- Targeting the NHEJ pathway could prove fruitful in combating therapy resistance and potentially achieving synthetic lethality in cancer cells.
- There are limited successes in the area of drug discovery for NHEJ so far and an urgent need to identify putative inhibitors.
- Advances in computational and structural biology have allow us step away from single components and examine PPIs as potential drug targets.

## Abbreviations

A-NHEJ: alternative nonhomologous end joining
APLF: aprataxin and PNK-like factor
ATP: adenosine triphosphate
BRCA: breast cancer gene
C-NHEJ: canonical nonhomologous end joining
cryo-EM: cryo electron microscopy
DNA-PK: DNA protein kinase
DNA-PKcs: DNA protein kinase catalytic subunit
HCC: hepatocellular carcinoma
HLH: helix-loop-helix
HNSCC: head and neck squamous cell carcinomas
HPV: Human Papilloma Virus
HR: homologous recombination
LigIV: DNA Ligase IV
MMEJ: microhomology-mediated end joining
NHEJ: non-homologous end joining
NMR: nuclear magnetic resonance
NSCLC: non-small cell lung carcinoma
PARP: poly (ADP-ribose) polymerase
PARPi: poly (ADP-ribose) polymerase inhibitor
PAXX: paralog of XRCC4 and XLF
PFDA: perfluorodecanoic acid
PI3: phosphoinositide 3
PI3K: phosphoinositide 3 kinase
PIKK: phosphatidylinositol 3-kinase-related kinase
PNKP: polynucleotide kinase/phosphatase
PPI: protein–protein interaction
SAXS: small angle X-ray scattering
TCM: Traditional Chinese Medicine Library
TDP1: tyrosyl DNA phosphodiesterase 1
XLF: XRCC4-like factor
XRCC4: X-ray repair cross-complementing protein 4
